# Hemophagocytic lymphohistiocytosis and thrombotic microangiopathy after parvovirus B19 infection and renal transplantation: a case report

**DOI:** 10.1186/s12882-021-02538-0

**Published:** 2021-10-12

**Authors:** C. J. Steffen, N. Koch, K. U. Eckardt, K. Amann, E. Seelow, A. Schreiber

**Affiliations:** 1 Department of Nephrology and Medical Intensive Care, Charité – Universitätsmedizin Berlin, Berlin, Germany; 2 Department of Nephropathology, Friedrich-Alexander University, Erlangen, Nürnberg, Germany

**Keywords:** Hemophagocytic Lymphohistiocytosis (HLH), Atypical hemorrhagic uremic syndrome (aHUS), Parvovirus B19, Case report

## Abstract

**Background:**

Hemophagocytic lymphohistiocytosis (HLH) is a rare and life-threatening disease characterized by hyperactivation of the immune system that causes hypercytokinemia and potentially multi organ failure. HLH can occur in patients with underlying rheumatic or autoinflammatory disorders. Additionally, HLH can develop in patients during infections or malignancies without a known genetic predisposition.

**Case presentation:**

We herein report a patient, who presented with fever, both acute kidney and liver injury, anemia, thrombocytopenia and HSV stomatitis. HLH was diagnosed based on clinical criteria and qPCR revealed an acute parvovirus B19 infection as potential underlying infectious trigger. Treatment was started with both IVIG and dexamethasone. Subsequently, kidney biopsy demonstrated TMA.

**Conclusions:**

In rare cases both HLH and aHUS can occur simultaneously in a patient as a consequence of viral infections. Insights from this unusual case might help physicians understand this complex symptom constellation.

## Background

Hemophagocytic lymphohistiocytosis (HLH) is a rare clinical condition (1/800000 adults per year, 1–10/Million children per year) with excessive immune activation and inflammation, eventually leading to multi organ failure. It can be divided into primary (genetic) forms which mostly affect children and secondary forms which are subclassified as viral, autoimmune, or paraneoplastic [1, 2].

Thrombotic microangiopathies (TMA) are a group of disorders characterized by microthrombi causing microangiopathic hemolytic anemia, thrombocytopenia and ischemic tissue injury [3]. TMAs encompass primary forms which occur spontaneously and secondary forms which develop in the context of pregnancy, autoimmune disease, malignancy, infection, malignant hypertension, bone marrow transplantation or the use of certain medications [4].

Here we report a case of simultaneous HLH and TMA after acute infection with parvovirus B19 (PVB19) in a kidney transplant recipient.

## Case presentation

In September 2020, a 50-year-old female was admitted to our hospital with PCR-diagnosed HSV-1 stomatitis and refractory anemia. The patient had received a cadaveric donor kidney transplantation for kidney failure due to IgA vasculitis in 2007 and was on triple immunosuppressive therapy including prednisolone (2.5 mg daily), mycophenolate mofetil (1500 mg daily) and cyclosporine A (100 mg daily). Laboratory testing on admission showed chronic allograft dysfunction (creatinine 3.1 mg/dl), anemia (Hb 5.9 g/dl) with low reticulocytes (32/nl) and low markers of systemic inflammation (CRP 1.9 mg/l, ferritin 324 μg/l). Transferrin saturation, vitamin B12 and folic acid levels, cyclosporine A through concentrations (48 ng/ml) as well as haptoglobin were within the normal range. There was no clinical sign of blood loss. Serum PCR-testing was negative for HSV-1/2, varicella zoster, CMV, EBV, HIV and parvovirus B19 (PVB19) respectively. A nasopharyngeal swab for SARS-CoV-2 was negative.

The patient was initially treated with intravenous acyclovir for 14 days, and immunosuppression was reduced by pausing mycophenolate mofetil and reducing cyclosporine A. Reticulocytes and hemoglobin levels recovered consequently and stomatitis resolved.

Eleven days after admission the patient suddenly developed fever up to 40 °C, low oxygen saturation (92%) and a cough. The lab results indicated acute on chronic allograft dysfunction (creatinine 4.1 mg/dl), acute hepatitis (ASAT 249 U/l, ALAT 300 U/l, bilirubin 4.1 mg/dl), moderately elevated CRP (29 mg/l) and an extremely high ferritin level (20,659 μg/l). Additionally, the patient showed thrombocytopenia (92/μl), elevated LDH (1170 U/l) and reduced haptoglobin (< 0.1 g/l), elevated d-dimer (8.8 mg/l), elevated soluble IL-2 receptor (12,458 IU/ml), elevated triglycerides (255 mg/dl) and low fibrinogen (1.0 g/l), reduced C3 and C4 (620 and 140 mg/l respectively). Coombs test was negative and a blood smear was negative for schistocytes. A chest CT scan ruled out pneumonia and pulmonary embolism. Abdominal ultrasound revealed ascites and moderate splenomegaly. Multiple urine and blood cultures did not show bacterial infection. A second PCR for SARS-CoV-2 was negative.

The diagnosis of hemophagocytic lymphohistiocytosis (HLH) was made based on the patient’s extremely high ferritin levels, presence of 6 out of 8 HLH diagnostic criteria and an HScore of 269 indicating a 99% probability of HLH. Treatment with dexamethasone 20 mg daily was started on day 13. Bone marrow aspiration and biopsy were conducted on day 14 and revealed no pathological findings (no evidence of HLH).

Fever subsided 1 day after starting dexamethasone, kidney function improved, and the levels of ferritin, transaminases and bilirubin decreased.

PCR for hepatitis B, C, D and E as well as for EBV and CMV were negative. Repeated PCR testing for PVB19 revealed > 10 million copies/ml. Treatment with intravenous immunoglobulins (100 g cumulative) was started over a course of 3 days to address the PVB19 infection. Dexamethasone dose was tapered to 8 mg on the day of discharge. PCR testing of the bone marrow sample revealed > 9 million copies/ml of PVB19. Serologic testing of blood samples was positive for IgM and negative IgG suggesting primary infection with PVB19.

In the following days the patient’s condition further improved, the viral load fell to 16,400 copies/ml at the time of discharge and ferritin levels decreased to 298 μg/l.

**Fig. 1:**
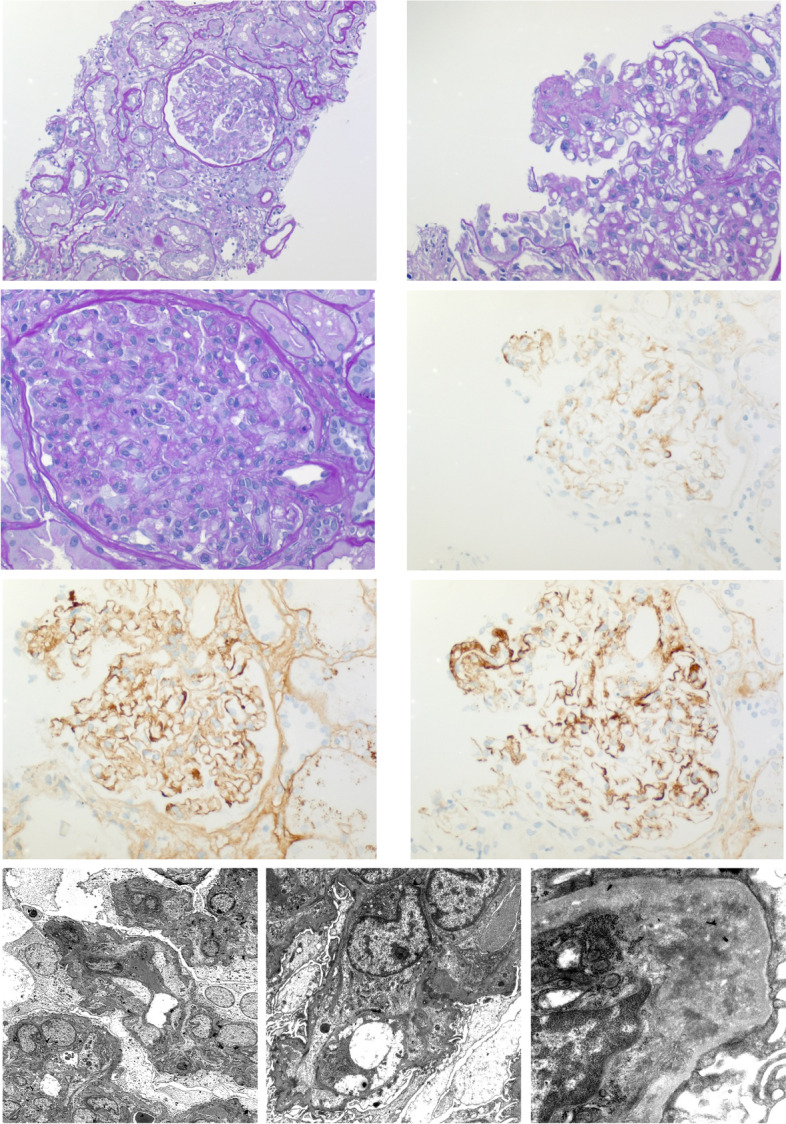
Characteristic histological findings in the renal biopsy. **A**-**C**: **A** Tubular atrophy and interstitial fibrosis with mild lymphoplasmacellular interstitial inflammation and acute tubular damage; no peritubular capillaritis, no eosinophils. **B**,**C**: Glomerular thrombotic microangiopathy (TMA) with swelling of endothelia cells, lumen obliteration, influx of inflammatory cells, some double contours of the basement membrane and some fibrin within glomerular capillaries (B, arrow). PAS staining, magnifications × 10 (**A**), × 20 (**B**) and × 40 (**C**). **D**-**F** Immunohistochemical findings: Granular mesangiocapillary immune deposits of IgA (**D**), IgG (**E**) and most prominently C3c (**F**) indicating a parainfectious glomerulonephritis. **G**-**J** Electron micrographs of different magnification showing the characteristic ultrastructural changes in thrombotic microangiopathy, e.g. endothelial cell swelling with subendothelial cleft formation and some podocyte damage. No specific osmiophilic deposits, no fibrils. **G**: × 2000, **H**: × 5000, **J**: × 8000

On day 35, the patient was re-admitted after a hypertensive crisis and a suspected seizure. Magnetic resonance imaging of the brain (including diffusion weighted imaging and fluid-attenuated inversion recovery sequences) as well as EEG did not show any pathological findings (e.g. posterior reversible leukoencephalopathy syndrome, intracranial hemorrhage). Laboratory testing showed worsening hemolysis, thrombocytopenia, complement consumption, schistocytes of 0.1% and further increasing albuminuria (UACR > 5 g/g). Ferritin, serum albumin levels and cyclosporine A through concentrations (40 ng/ml) were within the normal range and creatinine was stable at the patient’s previous baseline concentration. A renal allograft biopsy was performed, which showed glomerular TMA without vascular rejection, glomerulitis or peritubular capillaritis as well as tubular atrophy and 20% interstitial fibrosis (Fig. 1). ADAMTS13 diagnostic ruled out a TTP. On a follow up visit in our outpatient clinic on day 48 the patient was in good clinical condition. Ferritin was within the normal range and albuminuria was decreasing (UPCR 1.5 g/g), creatinine concentration was 3,5 mg/dl. Considering the gradual improvement we decided against therapeutic plasma exchange and complement inhibiting therapy Table 1.

**Table 1:** Laboratory findings during hospitalization

	Normal range	Day 1	Day 13	Day 14	Day 16	Day 36
Creatinine (mg/dl)	0.5–0.9	3.2	3.7	4.1	3.7	2.3
Urea (mg/dl)	17–48	137	70	143	166	110
CRP (mg/l)	< 0.5	4.4	21.5	29.2	19	2.6
Bilirubin (mg/dl)	< 1.2	0.2	4.1	2.1	0.8	0.5
Alanine aminotransferase (U/l)	< 31	12	300	201	121	85
Aspartate aminotransferase (U/l)	< 35	13	249	143	43	35
Lactate dehydrogenase (U/l)	135–250	203	679	974	763	454
Serum ferritin (μg/l)	13–150	324	2644	20,659	2563	194
Haptoglobin (g/l)	0.3–2.0	1.6	< 0.1	< 0.1	0.14	< 0.1
C3 (mg/l)	900–1800		550	620		470
C4 (mg/l)	100–400		130	140		80
Triglyceride (mg/dl)	< 200			211	255	
Fibrinogen (g/l)	1.6–4.0				1.1	
Hb (g/dl)	12.0–15.6	5.9	7.4	8.3	7.8	8.8
Leukocytes (n/nl)	3.9–10.5	3.4	3.2	3.1	6.5	6.2
Thrombocytes (n/nl)	150–370	302	187	162	92	91
Reticulocytes (n/nl)	25–105	32	169	145		212
Urinary Albumin/Creatinine (mg/gCrea)	< 20	169				5307

**Fig. 2:**
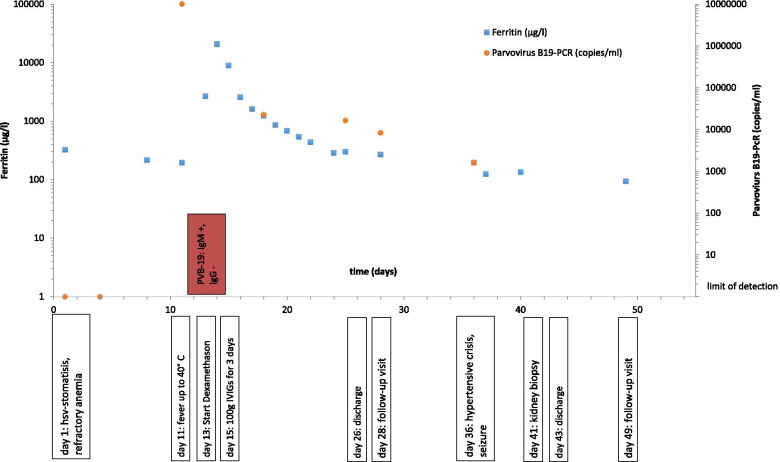
Clinical course, treatment milestones, ferritin levels and parvovirus B19 copies. Red dots indicate parvovirus B19 copies. The viral load was under the limit of detection on day 1 and day 3. Viral load declined following treatment with IVIGs. Serologic testing was negative for both IgM and IgG on day 1 and positive IgM on day 11, IgG stayed negative. Blue squares show ferritin levels

The clinical course, lab values and treatment are depicted in Fig. 2. Genetic screening did not reveal a pathogenic variant in complement genes. However the patient revealed to be homozygous for the known CFH-H3 (alias CFH_tgtgt_) risk haplotye as well as heterozygous carrier of the CFHR1*B risk allele.

## Discussion and conclusion

In the present case, suspicion for HLH was raised by otherwise unexplained multi organ failure and extremely high ferritin levels. The diagnosis of HLH was established by the 2004 diagnostic criteria [1]. The patient fulfilled 6 out of 8 criteria, 5 are considered sufficient for diagnosis (fever, splenomegaly, cytopenia of two lineages, hypertriglyceridemia and hypofibrigenemia, elevated sIL-2Receptor and hyperferritinemia). The absence of single clinical features, even a negative bone marrow biopsy, does not exclude HLH [1]. Other authors have proposed alternative diagnostic criteria such as the HScore, because the aforementioned HLH criteria were specifically developed for the pediatric population and other common laboratory findings such as elevated LDH, elevated liver enzymes, hepatic dysfunction and coagulopathy were not considered [5]. The HScore uses additional values such as immunosuppression, transaminases and LDH to calculate a probability score between 0 and 337 with values > 167 to classify > 90% of secondary HLH [5]. Our patient had an HScore of 267 indicating a 99% probability of HLH.

Glomerular TMA compatible with renal involvement was confirmed by renal allograft biopsy. Complement consumption, hypertension as well as seizures can be explained by aHUS, since EEG and MRI did not hint at an alternative diagnosis. Increasing albuminuria reflects glomerular damage. Cases of TMA triggered by PVB19 infection in both healthy and immunocompromised patients in the absence of an alternative cause have previously been reported [6–8]. The diagnosis of the PVB19 infection was made by both serum and bone marrow PCR testing. The results suggested a primary infection, indicated by the presence of specific IgM and the absence of specific IgG. When we tested the patient for viral infections on first admission, multiple PCR assays for PVB19 were negative. However other authors demonstrated that in some individuals PVB19 infection is only detectable in bone marrow samples whereas serum diagnostic can still be negative [9, 10]. In our case it is likely that PVB19 infection was already ongoing when the patient was admitted to our hospital.

Secondary HLH is typically a sequela of infections (usually EBV, but also CMV or HIV), rheumatic diseases (e.g. systemic lupus erythematosus) or malignancies [2]. A few cases of HLH due to PVB19 infection have previously been described, including one patient who had received renal transplantation [7, 10, 11].

Even though the incidence of acute kidney injury in HLH is high, the exact pathophysiologic mechanism of renal damage in HLH remains unclear and is probably multifactorial [12]. It has been proposed that PVB19 directly infects glomerular endothelium by using the same receptor (glycosphinoglipid globoside Gb4) used to infect erythroid progenitor cells. Subsequently it causes endothelial cell dysfunction or cell death leading to aHUS [6, 8]. Other studies suggest that aHUS and nephrotic proteinuria may have been caused primarily by HLH and only indirectly by PVB19 infection [13, 14]. A disease mechanism in HLH might be that high levels of inflammatory cytokines (particularly TNFα) can injure podocytes, thereby leading to glomerular collapse and tubular necrosis with subsequent proteinuria and renal dysfunction [13, 14]. Another recently proposed mechanism suggests coactivation of interferon gamma and complement activation as culprit in the evolution of TMA in patients with HLH [15]. In our patient the TMA should be classified as secondary aHUS triggered by parvovirus B19 Infection as well as by concomittant HLH. The risk haplotypes revealed by genetic screening display a high minor allele frequency in the general healthy population. In the absence of pathogenic complement variants they do not explain occurrence of aHUS. At most, they may have acted as additional disease modifiers in the setting of marked complement activation [16] .

A few limitations of our study are important to mention. First, a genetic testing for HLH risk haplotypes as well as functional complement assays (AH50; alternative pathway hemolytic assays) were not performed in this patient. Second, a long-term follow up for several years is still missing, therefore we cannot evaluate the likelihood of HLH relapses.

Treatment of HLH secondary to PVB19 infection in a transplant patient poses a challenge to balance immunosuppressive therapy to both address HLH and to fight PVB19 infection. In the present case, treatment with dexamethasone was initiated when HLH was suspected. After a positive PCR result for PVB19, intravenous immunoglobulins were added. Mycophenolat mofetil was discontinued and cyclosporine was reduced to a trough level of 50 ng/ml. In contrast to current recommendations for secondary HLH, we decided to taper dexamethasone relatively fast and did not prescribe etoposide [17]. We assumed PVB19 to be the underlying cause of aHUS. In summary, we observed the rare combination of HLH and TMA associated with parvovirus B19 that escaped initial PCR testing.

## Data Availability

The datasets used in this study are not publicly accessible because they contain identifying patient data. They can be provided by the corresponding author on reasonable request.

## Abbreviations

HLH: Hemophagocytic Lymphohistiocytosis
aHUS: Atypical hemorrhagic uremic syndrome
TMA: Thrombotic microangiopathy
PVB19: Parvovirus B19
